# Using machine learning algorithms for predicting cognitive impairment and identifying modifiable factors among Chinese elderly people

**DOI:** 10.3389/fnagi.2022.977034

**Published:** 2022-08-11

**Authors:** Shuojia Wang, Weiren Wang, Xiaowen Li, Yafei Liu, Jingming Wei, Jianguang Zheng, Yan Wang, Birong Ye, Ruihui Zhao, Yu Huang, Sixiang Peng, Yefeng Zheng, Yanbing Zeng

**Affiliations:** ^1^Tencent Jarvis Lab, Shenzhen, China; ^2^Institute of Mental Health, Peking University, Beijing, China; ^3^Institute of Psychology, Chinese Academy of Sciences, Beijing, China; ^4^Department of Psychology, University of Chinese Academy of Sciences, Beijing, China; ^5^Tencent Healthcare, Shenzhen, China; ^6^School of Public Health, Capital Medical University, Beijing, China

**Keywords:** cognitive impairment, machine learning, risk factor, intervention, elderly

## Abstract

**Objectives:** This study firstly aimed to explore predicting cognitive impairment at an early stage using a large population-based longitudinal survey of elderly Chinese people. The second aim was to identify reversible factors which may help slow the rate of decline in cognitive function over 3 years in the community.

**Methods:** We included 12,280 elderly people from four waves of the Chinese Longitudinal Healthy Longevity Survey (CLHLS), followed from 2002 to 2014. The Chinese version of the Mini-Mental State Examination (MMSE) was used to examine cognitive function. Six machine learning algorithms (including a neural network model) and an ensemble method were trained on data split 2/3 for training and 1/3 testing. Parameters were explored in training data using 3-fold cross-validation and models were evaluated in test data. The model performance was measured by area-under-curve (AUC), sensitivity, and specificity. In addition, due to its better interpretability, logistic regression (LR) was used to assess the association of life behavior and its change with cognitive impairment after 3 years.

**Results:** Support vector machine and multi-layer perceptron were found to be the best performing algorithms with AUC of 0.8267 and 0.8256, respectively. Fusing the results of all six single models further improves the AUC to 0.8269. Playing more Mahjong or cards (OR = 0.49,95% CI: 0.38–0.64), doing more garden works (OR = 0.54,95% CI: 0.43–0.68), watching TV or listening to the radio more (OR = 0.67,95% CI: 0.59–0.77) were associated with decreased risk of cognitive impairment after 3 years.

**Conclusions:** Machine learning algorithms especially the SVM, and the ensemble model can be leveraged to identify the elderly at risk of cognitive impairment. Doing more leisure activities, doing more gardening work, and engaging in more activities combined were associated with decreased risk of cognitive impairment.

## Introduction

Population aging is an important global public health issue. Cognitive decline is a natural process and is considered one of the most frightening aspects of aging (Ballard et al., 2011). Cognitive decline may develop into cognitive impairment. With improvements in life expectancy and an increasingly aging population, there will be a large population of the elderly with a high risk of cognitive impairment (Karlamangla et al., 2009). Serious cognitive impairment can lead to poor health of the elderly, which also exerts an enormous toll on their families and society (Langa et al., 2008; Hao et al., 2018). Elderly people with mild cognitive impairment may experience cognitive dysfunction, which may progress to dementia or Alzheimer’s disease (Zhang et al., 2019). Given the impact of dementia, the World Health Organization regards dementia prevention strategies as a public health priority (World Health Organization, 2017). Therefore, we would like to explore whether we could early identify individuals at risk of cognitive impairment and accordingly carry out an effective intervention in the community.

The gradual cognitive decline is common in late life. Due to the lack of effective treatment for dementia, prevention, and early identification are essential. As shown in a UK study, effective interventions for potentially modifiable risk factors of dementia would save £1,863 billion annually, and reduce dementia prevalence by 8.5% (Mukadam et al., 2020). The causative pathways that result in cognitive impairment are multifactorial and remain unclear. Although cognitive function is strongly associated with biological changes in the brain during aging, studies have assessed the role of genetics, psychosocial, and biochemical factors.

Several epidemiological studies have reported the association of social determinants of health and the risk of cognitive impairment, including educational levels, marital status, socioeconomic status, and residence (Håkansson et al., 2009; Mukadam et al., 2019). However, these factors about social determinants of health are not easily changeable. Some research revealed that lifestyle factors, including unhealthy diet, smoking, and lack of physical exercise, are associated with cognitive impairment (Anttila et al., 2004; Geda et al., 2012; Mottaghi et al., 2018). Physical diseases, including cardiovascular risk factors, hearing impairment, and tooth loss, are also related to cognitive impairment (Virta et al., 2013; Mukadam et al., 2019). In addition, previous studies found psychological factors and poor activities of daily living (ADL) increased cognitive impairment risk (Fauth et al., 2013). Some studies have evaluated the effects of multiple lifestyle factors in the Chinese elderly (Zhang et al., 2019; Mao et al., 2020; Qian et al., 2020; Li et al., 2021). For example, Qian et al. (2020) conducted a cross-sectional study in Suzhou. The study showed that almost all combinations of factors had a significant negative association with the risk of cognitive impairment, except the combination of tea consumption and siesta. A cross-sectional study by Zhang et al. (2019) described changes in cognitive function in the Chinese elderly from 2005 to 2014 and explored several risk factors; however, the study only included elderly individuals who survived from 2005 to 2014. Besides, a coarse binary quantification (Yes/No) of lifestyle factors was used in that study. Another study focused on leisure activities and found a greater frequency of watching TV or listening to the radio, reading books or newspapers, and playing Mahjong or cards may decrease the risk of cognitive impairment (Mao et al., 2020). However, few studies focused on the effects of behavior change with cognitive impairment. Studies in Korea showed that continuous physical activity and its relation to cognitive function (Song and Park, 2022) and found promotion of participation in religious organizations, friendship organizations, and family/school reunions (only for older persons) may help preserve cognitive function in individuals aged 45 years or older (Choi et al., 2016). Therefore, we are wondering whether the change of behaviors is associated with cognitive impairment in the Chinese population. Specificity, whether activities with Chinese characteristics (e.g., playing Mahjong) are associated with cognitive impairment in the elderly.

Machine learning techniques have been used for classification, which can help in revealing potential hidden dependencies between factors and outcomes (Bratić et al., 2018). To our knowledge, this study is among the first in developing a machine learning framework for identifying Chinese elderly people at risk of cognitive impairment (Wang B. et al., 2020; Hu M. et al., 2021). Few studies have shown that demographics, genetic factors, brain imaging, and blood biomarkers have the potential to inform a healthy person’s likelihood of progression to mild cognitive impairment (Chang et al., 2021; Stonnington et al., 2021). However, the cost of invasive tests and brain imaging is relatively high. And these models are designed to identify risk factors for cognitive impairment/dementia among people with normal cognition at baseline. Moreover, most of these factors are not modifiable, therefore do not allow us to intervene in advance. Examining predictors generated by a predictive model can deliver important information about modifiable risk factors to the public. Being able to predict cognitive decline would be a step forward in selecting people for therapy or prevention. To fill these gaps, we expect to identify the effective behavior alterations to prevent cognitive impairment from the perspective of public health.

Therefore, based on national survey data focused on the oldest old who had rarely been examined from 2002 to 2014, this study aimed to build a prediction model with machine learning algorithms to early identify the elderly at risk for cognitive impairment 3 years in advance and to further examine multi-influencing factors associated with lifestyle behavior simultaneously on cognitive decline.

## Methods

### Data sources

In this study, we used data from the CLHLS, a large population-based longitudinal survey of centenarians, nonagenarians, and octogenarians (Chinese Longitudinal Healthy Longevity Survey (CLHLS), 2017). It was based on a randomly recruited set of elderly Chinese adults aged 65 and above from half of the cities in 23 out of 31 provinces of mainland China, whose populations together constitute about 85% of the total population in China (Shen and Zeng, 2014). The survey began in 1998, and examinations are carried out every 2–3 years. Further details of the CLHLS sampling design, response rates, questionnaire validity, and data quality were published extensively elsewhere (Yi et al., 2001; Zeng, 2012).

### Predictor variables

Candidate variables were assessed based on demographic characteristics and established risk factors. Detailed information is presented in Table 1 and Supplementary Table 1. A total of 26 variables were selected as potential features from six categories, namely demographic, psychological, lifestyle, social/entertainment activities, ADL, and chronic disease.

**Table 1:** Demographic characteristics of the participants between groups with and without cognitive impairment (CI) 3 years later (*N* (%)).

**Variables**	**Without CI 3 years later (N = 11,081)**	**With CI 3 years later (N = 1,199)**	**P value**
**Age (years)**			<0.001
–79	4,776 (43.10)	78 (6.51)	
80–89	3,132 (28.26)	231 (19.27)	
90–99	2,436 (21.98)	501 (41.78)	
100–	737 (6.65)	389 (32.44)	
**Gender**			<0.001
Female	5,438 (49.07)	852 (71.06)	
Male	5,643 (50.93)	347 (28.94)	
**Education(years)**			<0.001
0	5,625 (50.76)	938 (78.23)	
1-6	3,897 (35.17)	205 (17.10)	
≥ 7	1,559 (14.07)	56 (4.67)	
**Marital status**			<0.001
Without spouse	5,414 (48.86)	970 (80.90)	
With spouse	5,667 (51.14)	229 (19.10)	
**Economy status**			<0.001
Normal	7,658 (69.11)	776 (64.72)	
Rich	1,902 (17.16)	200 (16.68)	
Poor	1,521 (13.73)	223 (18.60)	
**Residence type**			<0.001
City	1,628 (14.69)	116 (9.67)	
Rural	9,453 (85.31)	1,083 (90.33)	
**Co-residence**			<0.001
Alone	1,637 (14.77)	183 (15.26)	
With household member	9,239 (83.38)	966 (80.57)	
In a nursing home	205 (1.85)	50 (4.17)	

### Demographic factors

Demographic factors consisted of seven variables, including gender (male or female), age group (–79, 80–89, 90–99, or 100–), type of birthplace (urban or rural), co-residence (alone, with household members, or in a nursing home), educational levels (illiterate, 1–6 years, or ≥7 years), marital status (with or without a spouse), and self-rated economic status (rich, normal, or poor).

### Psychological factors

Psychological factors (Zhang et al., 2019) contained one numerical variable as depression score, which was calculated from seven questions, namely “Do you always look on the bright side of things?”, “Do you like to keep your belongings neat and clean?”, “Do you often feel fearful or anxious?”, “Do you often feel lonely and isolated?”, “Can you make your own decisions concerning your personal affairs?”, “Do you feel the older you get, the more useless you are?”, and “Are you as happy as when you were younger?”. The total score ranges from 7 to 35, with a lower score indicating better psychological status.

### Lifestyle

Lifestyle contained five factors, which were a current smoker (yes or no), current drinker (yes or no), exercise (yes or no), frequency of eating fruits (every day or almost every day, quite often, occasionally, rarely or never), and frequency of eating vegetables (every day or almost every day, quite often, occasionally, rarely or never).

### Social/entertainment activities

Social/entertainment activities contained seven variables, namely personal outdoor activities, garden work, reading newspapers/books, raising domestic animals, playing Mahjong and/or cards, watching TV and/or listening to the radio, and social activities (organized). All these variables were given values ranging from 1 to 5, with higher scores indicating higher frequency.

### ADL

ADL was measured by six questions reflecting the disability in bathing, dressing, toilet, indoor movement, continence, and eating. If one had difficulties in any of the six activities, the corresponding ADL label would be 1, otherwise 0.

### Chronic diseases

Chronic diseases contained four validated cognitive-impairment-related illnesses, namely hypertension, diabetes, stroke or cerebrovascular disease, and Parkinson’s disease (Luchsinger et al., 2007; Obisesan, 2009; Kalaria, 2012).

## Feature selection

Feature selection can reduce the complexity of the model without much loss of the total information. It also helps to increase the interpretability and accuracy of the model. Feature selection was performed using sequential forward floating selection (SFFS; Somol et al., 2010), which is a greedy search algorithm that searches for an optimal combination of features. During the process of feature selection, 3-fold cross-validation was performed to evaluate the accuracy of the current feature set. The stopping criteria for SFFS were defined as: (1) no increase in the AUC by 0.001 after 10 consecutive iterations, or (2) the predetermined maximum number of features has been reached. We first performed feature selection for each of the machine learning methods. Then we trained models with features selected by each of the models.

## Assessment of cognitive impairment

Cognitive function was measured using the Mini-Mental State Examination (MMSE), a frequently used screening instrument for global cognitive dysfunction. The questionnaire was adapted into the Chinese version and tested by previous pilot survey interviews (Gao et al., 2015; Zhang et al., 2019). The total score ranges from 0 to 30, which consists of 24 items within six dimensions: five items for orientation, three for registration, one for naming, five for attention and calculation, three for recall, and seven for language; A higher score indicates better cognitive function. Cognitive impairment was defined as the MMSE score below 18, which has been previously validated as an appropriate criterion (An and Liu, 2016; Gao et al., 2017).

## Study design

**Figure 1:**
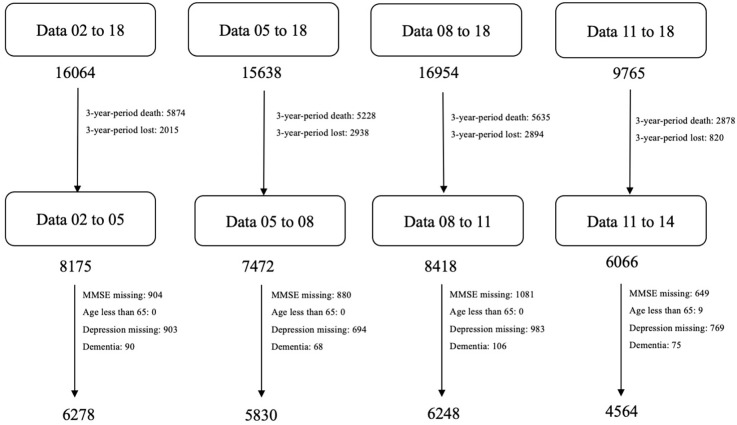
The inclusion and exclusion criteria of this study.

We first included four non-overlapping waves of 3-year survey data between 2002 and 2014. The detailed flow chart of participant selection is shown in Figure 1. All the participants were followed up every 3 years (a wave). After eliminating participants that died or were lost to follow-up in each wave, the numbers of the elderly for each wave were 8,175, 7,472, 8,418, and 6,066, respectively. Then, we included participants aged 65 years or above, and excluded participants diagnosed with dementia, or missing either MMSE or depression score at baseline. After that, four waves had sample sizes of 6,278, 5,830, 6,248, and 4,564, respectively. Finally, we combined data from four waves together, and a total of 22,920 records were eligible for analysis. As some individuals participated in the survey two or three times, 12,280 individuals were included.

**Figure 2:**
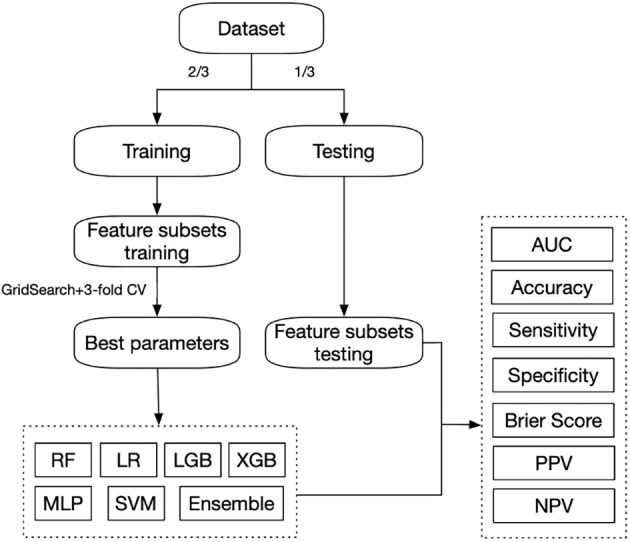
Schematic diagram of our prediction framework.

After combining four waves of data, the data were divided into 2/3 for training and 1/3 for testing. During parameter tuning for each model, grid search and 3-fold cross-validations were used to find the parameters of best performance in the training data. Six machine learning algorithms were trained, including extreme gradient boosting (XGboost), random forest (RF), logistic regression (LR), support vector machine (SVM), lightGBM (LGB), and multilayer perceptron (MLP). We also ensemble these six models by stacking. The model evaluation was performed using accuracy, area-under-curve (AUC), sensitivity, specificity, and the Brier score in the test data (Figure 2). AUC is an aggregated measure of the algorithm’s ability to discriminate outcome classes across all possible classification thresholds, and the Brier score measures the accuracy of prediction. A higher AUC or a lower Brier score indicates better prediction performance (Zhang et al., 2021).

## Dealing with unbalanced data

Since the data is unbalanced, the parameter “class_weight” is used to rebalance the distribution. In other words, the weight of the sample *τ* which belongs to class j is set to N_samples/(N(class_j)×(Nclasses) when calculating the loss in each machine learning model.

## Statistical analysis

Categorical variables were reported as numbers (proportions) and compared using a chi-square test or Fisher exact test. Continuous variables were presented as mean with standard deviation (SD). Of all observations, only two covariates contained missing values. The number (percentage) of missing values for co-residence and travel times was 28 (0.13%) and 31 (0.14%), respectively. We filled the missing categorical values (i.e., co-residence) with the mode of the distribution. Mean values were used to perform the imputation of missing numerical values (i.e., travel times). Numeric features were then standardized into zero mean and unit variance.

The frequency of eating fruits, eating vegetables, personal outdoor activities, gardening work, reading newspapers or books, raising domestic animals, playing Mahjong or cards, watching TV or listening to the radio was categorized into binary variables. For the frequency of eating fruits, we classified it into the value 0 if the person never or rarely ate fruits; otherwise, it was classified into 1. For the frequency of eating vegetables, we classified it into 0 or 1, with 0 referring to the person who never or rarely or sometimes ate vegetables and 1 otherwise. As playing Mahjong or cards, the frequency was assigned value 1 if the person did these things every day or almost every day, otherwise 0. The frequency of garden work, reading books/newspapers, watching TV or listening to the radio was assigned value 0 if the person never or rarely did these activities, otherwise was assigned 1.

To enable future point-of-care behavior intervention and consider the model interpretability, logistic regression models were used. Firstly, we assessed the association between selected features and the outcome. Then, we included the change of the features into the model considering the effect of life behavior change in the three-year period. We defined the behavior change as “doing less” or “doing more”, referring to the decrease or increase of the frequency of doing the specific activity. We further classified the positive behavior change into “a little bit more” or “much more”. “A little bit more” refers to the frequency of doing the specific activity increasing one or two degrees. For example, the individual never or rarely did an activity three years ago but now does the activity sometimes or monthly. “Much more” refers to the frequency of doing the specific activity increasing three or four degrees. For example, the individual never or rarely did an activity three years ago but now does the activity weekly or daily. Besides, we defined the combination of lifestyle behavior, refers to the number of six modifiable activities (personal outdoor activities, gardening work, reading newspapers or books, raising domestic animals, playing Mahjong or cards, watching TV, or listening to the radio). The number of the combination of the behavior change was defined as “being less” or “being more”, referring to the decrease or increase of the number of the combination lifestyle behavior. We further classified the number of changes into “being less”, “being a little bit less”, “being a little bit more”, and “being more”. “Being less” refers to the number between −6 to −4, “Being a little bit less” refers to the number between −3 to −1, “being a little bit more” and “being more” refers to 1–3, and 4–6, respectively. Odds ratio (OR) and 95% confidence intervals (CI) were estimated with the logistic regression models. Machine learning algorithms were trained and evaluated using scikit-learn and seaborn in Python (3.6.5). The MLP model was developed with PyTorch 1.6.0.

## Results

### Baseline characteristics

A total of 6,278, 5,830, 6,248, and 4,564 elderly people aged 65 or older participated in the baseline wave of 2002–2005, 2005–2008, 2008–2011, and 2011–2014, respectively. We divided the individuals into two groups: with or without cognitive impairment 3 years later. Participants with cognitive impairment 3 years later tended to be older; female; without spouse; more likely to smoke or drink alcohol; less likely to exercise; less likely to eat fruits; less likely to do garden work; less likely to read newspapers/books; less likely to watch TV and/or listen to the radio and having a higher rate of hypertension. The descriptive statistics of the two groups were presented in Table 1 and Supplementary Table 1.

### Prediction models

**Figure 3:**
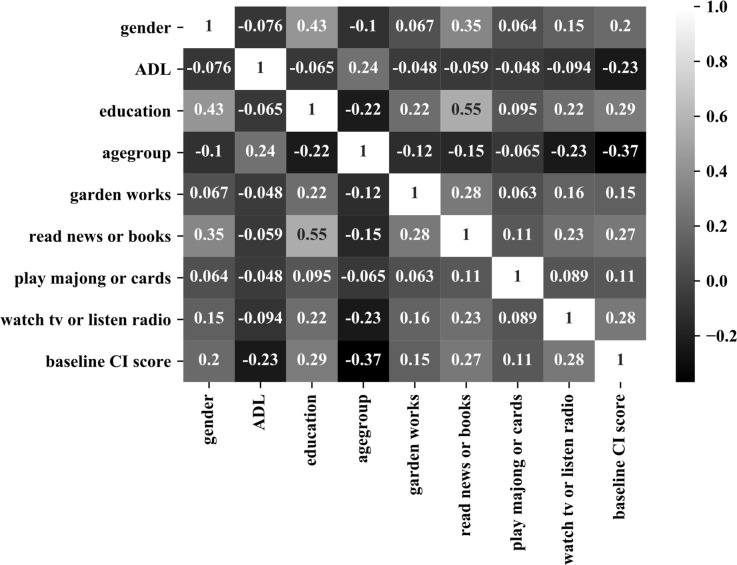
Pearson correlation among the selected features.

Table 2 shows the selected features by five machine learning models; as all features were fed into the network, there was no feature selection in the MLP model. A total of 12 unique features were selected by at least one model. Age group was selected by all five models, followed by education level, watching TV/listening to the radio, baseline MMSE, which were selected by four models. Figure 3 shows the Pearson correlation among the features.

**Table 2:** Selected features by each model.

**Model**	**Selected features**
Logistic Regression (9)	age group, education level, gender, ADL, garden works, reading newspapers or books, playing Mahjong or cards, watching TV/listening to the radio, baseline MMSE
Support Vector Machine (4)	age group, reading newspapers or books, playing Mahjong or cards, watching TV/listening to the radio
Random Forest (9)	age group, education level, co-habitation, exercise, ADL, marital status, garden works, watching TV/listening to the radio, baseline MMSE
LightGBM (6)	age group, education level, marital status, garden works, reading newspapers or books, baseline MMSE
XGBoost (6)	age group, education level, co-habitation, ADL, watching TV/listening to the radio, baseline MMSE

Results of the algorithm performance are shown in Table 3 and the best parameters for models are shown in Supplementary Table 2. The confusion matrix for each model is shown in Supplementary Table 3. In the test set, ROC curves revealed that the SVM model had the best performance, with an AUC of 0.8267. The AUC of MLP, LR, and LGB was 0.8256, 0.8248, and 0.8238, respectively. Fusing the results of all six models further improves the AUC to 0.8269. The model of RF performed well in sensitivity, with a value of 0.8256. The model of MLP and LR performed well in specificity, with a value of 0.7556 and 0.7417, respectively.

**Table 3:** Performance of machine learning models in the test set with features selected by logistics regression.

**Algorithm**	**Accuracy**	**AUC**	**Sensitivity**	**Specificity**	**Brier Score**	**PPV**	**NPV**
Logistic Regression	0.7429	0.8248	0.7549	0.7417	0.1775	0.2198	0.9691
Support Vector Machine	0.7303	0.8267	0.7699	0.7265	0.0692	0.2134	0.9704
Random Forest	0.6589	0.8057	0.8256	0.6428	0.1894	0.1822	0.9745
LightGBM	0.7062	0.8238	0.8000	0.6972	0.1819	0.2030	0.9731
XGBoost	0.7283	0.8234	0.7669	0.7246	0.1955	0.2116	0.9699
Multi-layer Perceptron	0.7540	0.8256	0.7368	0.7556	0.1681	0.2252	0.9675
Fusion	0.7236	0.8269	0.7684	0.7192	0.1804	0.2087	0.9699

### Association between features and cognitive impairment

As the prediction performance was similar for the models, we used LR to analyze the association between selected features and cognitive impairment. Using SFFS, nine features were selected to be incorporated in the models, including gender, age group, education level, ADL, garden works, reading newspapers or books, playing Mahjong or cards, watching TV/listening to the radio, and baseline MMSE. Educational level was a predictive factor of cognitive impairment. Compared with illiterate, individuals with an education of 1–6 years or 7 years above had a lower risk of cognitive impairment (OR = 0.66, 95% CI: 0.58–0.77; OR = 0.60, 95% CI: 0.47–0.77, respectively). Compared with individuals without normal ADL, those with poor ADL were 1.25 times more likely to develop cognitive impairment. The individuals who doing garden works (OR = 0.75, 95% CI: 0.63–0.89), reading newspapers or books (OR = 0.80, 95% CI: 0.67–0.97), playing Mahjong or cards (OR = 0.69, 95% CI: 0.53–0.90), watching TV or listening to the radio (OR = 0.90, 95% CI: 0.89–0.90) decreased the risk of cognitive impairment compared with those who rarely or never do these activities (Supplementary Table 4).

Table 4 illustrates the results of the association between longitudinal behavior change and cognitive impairment. Compared with Compared with those whose behavior did not change, the associations of playing less Mahjong or cards (OR = 1.27,95% CI: 1.06–1.51), doing fewer garden works (OR = 1.36, 95% CI: 1.04–1.77), reading fewer newspapers or books (OR = 4.18, 95%CI: 2.55–6.83), watching less TV or listening to less radio (OR = 2.27,95% CI: 1.99–2.60) were more likely to develop cognitive impairment after adjustment for the baseline behavior status. Regarding the number of combinations of behavior changes, the results showed that the decreased number of the combination change of lifestyle behavior was associated with the risk of cognitive impairment (shown as model 1 in Table 4). We further explored the degree of behavior change and its impact on the outcome (model 2 in Table 4). Compared with individuals who did not change the frequency of playing Mahjong or cards, those who played a little bit more or played much more decreased the risk of developing cognitive impairment (OR = 0.58,95% CI: 0.42–0.81; OR = 0.37,95% CI: 0.24–0.56, respectively). Similar results were found in doing garden works. As in watching TV or listening to the radio, we only found watching or listening much more had a protective effect on cognitive impairment (OR = 0.52,95% CI: 0.44–0.63). As for the degree of the change in the combination with lifestyle behavior, we found the OR of being less was 8.47 (95% CI: 4.81–14.91) and was 0.06 (95% CI: 0.01–0.47) for being more.

**Table 4:** The association of lifestyle change with cognitive impairment.^*^

**Variable**	**N (%)**	**OR (95% CI)**	**P values**
		**Model 1**	
**Change in playing Mahjong or cards**			
No change	16,950 (73.95)	REF	
Playing less	3,341 (14.58)	1.27 (1.06, 1.51)	0.009
Playing more	2,629 (11.47)	0.49 (0.38, 0.64)	<0.001
**Change in garden works**			
No change	16,405 (71.58)	REF	
Doing less	3,315 (14.46)	1.36 (1.04, 1.77)	0.026
Doing more	3,200 (13.97)	0.54 (0.43, 0.68)	<0.001
**Change in reading newspapers or books**			
No change	17,199 (70.04)	REF	
Reading less	3,166 (13.81)	4.18 (2.55, 6.83)	<0.001
Reading more	2,555 (11.15)	0.79 (0.61, 1.03)	0.085
**Change in watching TV or listening to the radio**			
No change	12,606 (55.00)	REF	
Watching or listening less	5,503 (24.01)	2.27 (1.99, 2.60)	<0.001
Watching or listening more	4,811 (20.99)	0.67 (0.59, 0.77)	<0.001
**Change in combination of lifestyle behavior**			
No change	7,255 (31.65)	REF	
Being less	9,036 (39.43)	1.66 (1.44, 1.91)	<0.001
Being more	6,629 (28.92)	0.55 (0.46, 0.64)	<0.001
			**Model 2**
**Change in playing Mahjong or cards**			
No change	16,950 (73.95)	REF	
Playing less	3,341 (14.58)	1.29 (1.08, 1.53)	0.005
Playing a little bit more	1,499 (6.54)	0.58 (0.42, 0.81)	0.001
Playing much more	1,130 (4.93)	0.37 (0.24, 0.56)	<0.001
**Change in garden works**			
No change	16,405 (71.58)	REF	
Doing less	3,315 (14.46)	1.44 (1.11, 1.87)	0.006
Doing a little bit more	1,003 (4.38)	0.61 (0.43, 0.87)	0.006
Doing much more	2,197 (9.59)	0.47 (0.36, 0.62)	<0.001
**Change in watching TV or listening to the radio**			
No change	12,606 (55.00)	REF	
Watching or listening less	5,503 (24.01)	2.51 (2.19, 2.87)	<0.001
Watching or listening a little bit more	2,569 (11.21)	0.91 (0.76, 1.09)	0.314
Watching or listening much more	2,242 (9.78)	0.52 (0.44, 0.63)	<0.001
**Change in combination of lifestyle behavior** ^**^			
No change	7,253 (31.65)	REF	
Being less	170 (0.74)	8.47 (4.81, 14.91)	<0.001
Being a little bit less	8,868 (38.69)	2.21 (1.93, 2.52)	<0.001
Being a little bit more	6,521 (28.45)	0.51 (0.44, 0.59)	<0.001
Being more	108 (0.47)	0.06 (0.01, 0.47)	0.007

^*^Adjustment for gender, education, age group, baseline MMSE, baseline ADL, baseline garden works, baseline reading newspapers or books, baseline playing Mahjong or cards, baseline watching TV or listening to the radio. Less: the frequency of doing the specific activity decreases. More: the frequency of doing the specific activity increases. A little bit more: the frequency of doing the specific activity increases one or two degrees. Much more: the frequency of doing the specific activity increases three or four degrees. ^**^Being less, being a little bit less, being a little bit more, and being more refers to the degree of change between −6 to −4, −3 to −1, 1 to 3, and 4 to 6, respectively.

## Discussion

In this study, we developed prediction models using machine learning algorithms to predict further cognitive impairment in 12,280 individuals with 22,920 records using variables obtained by questionnaires. Besides, our research focused on multiple modifiable risk factors simultaneously based on the prediction models. We found playing more Mahjong or cards, doing more garden work, watching TV/listening to the radio more are associated with decreased risk of cognitive impairment after 3 years.

In this article, we provided a fusion model as a simple tool for screening cognitive impairment. The findings have potential public health significance in the elderly. Given that cognitive impairment may be modifiable (Sha et al., 2022), our study could help the development of a new tool for the early identification of community-support needs, especially for relatively young group of the elderly people and those with currently normal cognitive function. Previous papers mostly used conventional statistical methods such as Cox proportional hazards regression models (Zhou et al., 2020). However, we used machine learning which may pave a path towards early preventive health care decision support for cognitive impairment risk identification with potential benefits for prevention (Wiemken and Kelley, 2020). The Cox model relies on the assumption of proportional hazards across different covariates (Kuitunen et al., 2021). Compared with the Cox model, machine learning models used in this paper would reflect a complex relationship among the various risk factors. Therefore, the model can achieve higher predictive accuracy than Cox regression models. Kim et al. (2019) compared the performance between Cox models and machine learning models and found deep learning algorithms using time-series data could be an accurate and cost-effective method to predict dementia. For comparison, the study by Hu et al. (2021) used the same data source as ours and ecluded individuals with abnormal MMSE at baseline, and included four features to predict cognitive impairment in 6,718 elderly people. In this study, we included participants with abnormal MMSE at baseline in the model as we found the score of MMSE in some individuals improved 3 years later, which might have a wider range of applications in preventive health care. Furthermore, we also tried deep learning prediction methods and model ensembling to evaluate the performance. Wang Z. et al. (2020) built a decision tree model for 625 elderly people and quantitatively measured the importance of predictive variables including social engagement, a high-fat diet, tea-drinking, hobbies, living conditions, and smoking. However, the outcome used in that study was the current cognitive status of the subject, not a future event.

Besides building a prediction model, we also integrated it with intervention strategies, which can be served for the policy management. Besides unmodifiable risk factors, we focused on longitudinal behavior change and the combination of lifestyle behaviors. From the report of the National Health Commission of the People’s Republic of China (2020), patients diagnosed with mild cognitive impairment are presented with recommendations regarding nonpharmacologic interventions in the community. We found routine behaviors including playing more Mahjong or cards, doing more garden works, reading more newspapers or books, watching more TV or listening to more radio were less likely to develop cognitive impairment after adjustment for the baseline behavior status. Especially, we found the risk of cognitive impairment decreased with the increased degree of change in playing Mahjong or cards and in garden works. Mahjong is a popular form of social entertainment for elderly people in China. The participants need to focus and coordinate visual, mental, and body activities. Zhang et al. (2020) found playing Mahjong for 12 weeks improved the executive function of elderly people with mild cognitive impairment. Playing Mahjong or cards can be classified into leisure activities. The underlying mechanism of the protective effects of leisure activities on cognitive function is not yet clear. The cognitive reserve hypothesis suggests that an engaged lifestyle may enable related neural networks to be more efficient or plastic, resulting in the postponement of dementia onset or less cognitive deterioration (Stern, 2012). Furthermore, loneliness was associated with decreased cognitive function over a 3-year follow-up period (Lara et al., 2019). Teh and Tey (2019) showed that active participation in playing Mahjong/cards can be an effective intervention against persistent loneliness. Besides leisure activities, we found gardening works had a protective effect on cognitive impairment. A 4-year longitudinal study indicated that individuals who continue to engage in fieldwork or gardening, reading books or newspapers have an increased chance of recovery from mild cognitive impairment to normal cognition (Shimada et al., 2019). Park et al. (2019) revealed a potential benefit of gardening activities for cognitive function in senior individuals. They found levels of brain-derived neurotrophic factor and platelet-derived growth factor were significantly increased after the gardening activity, which was brain nerve growth factors related to memory and cognitive function. Our findings support the previous results. In addition, we focused on the combination multiple of lifestyle behavior changes to observe the outcome and found the combination of more behavior changes was associated with a reduced risk of cognitive impairment. Therefore, we recommend the elderly try to engage in different kinds of activities. A similar result was found in Minnesota that engaging in a higher number of activities in late life was associated with a significantly reduced risk of incident mild cognitive impairment (Krell-Roesch et al., 2019), however, the questionnaire on activities engagement was only collected at baseline. The daily life behavioral interventions identified in this study are effective in preventive health care as these daily life behaviors are simple, low-cost entertainment activities, and easy to apply.

The strengths of our study include the population-based design and the large sample size. We expect this study could provide a risk estimation for cognitive impairment after 3 years in the community based on the current health status. Simultaneously, offering modifiable behaviors could help to slow the progression of the disease. Furthermore, we focused on the degree of behavior change and found modifiable risk factors related to healthy lifestyles and their optimization can slow the process of cognitive impairment. This study can benefit policymakers in aging countries such as China by providing effective and specific policy advice about community-based elderly care. However, our research has some limitations. First, although we utilized four waves of the data and used a cross-validation method to build models, the results need to be validated in another independent cohort. Second, some of the factors in this study were measured by self-reporting, which may result in information bias. However, self-reported information is easy to obtain in preventive health care. Third, future experimental research is needed to verify the impact of lifestyle behaviors on the physiological progress of cognitive impairment as the study did not include biological data. Fourth, we excluded participants with missing MMSE scores like other related works (Lv et al., 2019; Hu X. et al., 2021); however, we cannot determine the cause of missing in these people. It is possible that the main reason was severe cognitive impairment, and this might induce selection bias.

## Conclusions

Risk-predictive models may serve as a valuable tool to support assessing the risk of cognitive impairment in community-based preventive health care. SVM and ensemble models were found to be the best performing algorithm. Modifiable risk factors including doing more leisure activities, doing more gardening work, and participating in more activities combined were identified and suggested to slow the rate of cognitive decline.

## Data Availability Statement

All data used in this study were stored at https://opendata.pku.edu.cn and available upon request.

## Ethics Statement

The use of CLHLS data was approved by the Biomedical Ethics Committee of Peking University.

## Author Contributions

SW is the chief investigator for the study and is responsible for the study concept and design, and critical revision of the manuscript. WW and YL contributed to analysis and interpretation of the data and writing of the draft. XL, JW, and YH contributed to the analysis and interpretation of the data. BY and RZ contributed to the design of the questionnaire and the management and quality control of the cohort. JZ, SP, and YW contributed to the software and supervision. YZh and YZe contributed to revision and validation. All authors contributed to the article and approved the submitted version.

## Funding

This work was supported by the National Natural Science Foundation of China (71874147) and Research Center for Capital Health Management and Policy (2022JD01).
