# Supplementary material for: Using machine learning algorithms for predicting cognitive impairment and identifying modifiable factors among Chinese elderly people
Source: Front Aging Neurosci. 2022 Aug 11;14:977034. doi: 10.3389/fnagi.2022.977034 (PMC9407018; doi:10.3389/fnagi.2022.977034)
Supplement: Supplementary file 1 [file Data_Sheet_1.docx]

| Supplemental Table 1. Life behaviors and disease information between groups with and without cognitive impairment (CI) after 3 years. | | | |
| --- | --- | --- | --- |
| **Variables** | **Without CI 3 years later (N=11,081)** | **With CI 3 years later (N=1,199)** | ***P* value** |
|  | Mean (SD) | |  |
| **Travel times in recent 2 years** | 0.26 (1.50) | 0.08 (0.51) | <0.001 |
| **Psychological score** | 20.57 (2.60) | 20.73(2.76) | 0.041 |
|  | N (%) | |  |
| **ADL** |  |  | <0.001 |
| No | 10,173 (91.81) | 927 (77.31) |  |
| Yes | 908 (8.19) | 272 (22.69) |  |
| **Smoke** |  |  | <0.001 |
| No | 8,439 (76.16) | 1,020 (85.07) |  |
| Yes | 2,642 (23.84) | 179(14.93) |  |
| **Alcohol** |  |  | <0.001 |
| No | 8,421 (75.99) | 981 (81.82) |  |
| Yes | 2,660 (24.01) | 218 (18.18) |  |
| **Exercise** |  |  | <0.001 |
| No | 6,975 (62.95) | 898 (74.90) |  |
| Yes | 4,106 (37.05) | 301 (25.10) |  |
| **Eating fruits** |  |  | <0.001 |
| Rarely or never | 2,498 (22.54) | 382 (31.86) |  |
| Occasionally | 4,251 (38.36) | 469 (39.12) |  |
| Quite often | 2,985 (26.94) | 235 (19.60) |  |
| Everyday or almost everyday | 1,347 (12.16) | 113 (9.42) |  |
| **Eating vegetables** |  |  | <0.001 |
| Rarely or never | 195 (1.76) | 35 (2.92) |  |
| Occasionally | 883 (7.97) | 129 (10.76) |  |
| Quite often | 3,373 (30.44) | 389 (32.44) |  |
| Everyday or almost everyday | 6,630 (59.83) | 646 (53.88) |  |
| **Personal outdoor activities** |  |  | <0.001 |
| Rarely or never | 2,423 (21.87) | 428 (35.70) |  |
| Sometimes | 563 (5.08) | 74 (6.17) |  |
| Monthly | 409 (3.69) | 49 (4.09) |  |
| Weekly | 1,226 (11.06) | 142 (11.84) |  |
| Daily | 6,460 (58.30) | 506 (42.20) |  |
| **Garden work** |  |  | <0.001 |
| Rarely or never | 8,801 (79.42) | 1,085 (90.49) |  |
| Sometimes | 281 (2.54) | 24 (2.00) |  |
| Monthly | 202 (1.82) | 17 (1.42) |  |
| Weekly | 385 (3.47) | 17 (1.42) |  |
| Daily | 1,412 (12.74) | 56 (4.67) |  |
| **Reading newspapers/books** |  |  | <0.001 |
| Rarely or never | 7,970 (71.92) | 1,087 (90.66) |  |
| Sometimes | 418 (3.77) | 24 (2.00) |  |
| Monthly | 290 (2.62) | 15 (1.25) |  |
| Weekly | 665 (6.00) | 27 (2.25) |  |
| Daily | 1,738 (15.68) | 46 (3.84) |  |
| **Raising domestic animals** |  |  | <0.001 |
| Rarely or never | 7,122 (64.27) | 896 (74.73) |  |
| Sometimes | 301 (2.72) | 31 (2.59) |  |
| Monthly | 206 (1.86) | 23 (1.92) |  |
| Weekly | 466 (4.21) | 64 (5.34) |  |
| Daily | 2,986 (26.95) | 185 (15.43) |  |
| **Playing Mahjong or cards** |  |  | <0.001 |
| Rarely or never | 8,427 (76.05) | 1,056 (88.07) |  |
| Sometimes | 503 (4.54) | 31 (2.59) |  |
| Monthly | 397 (3.58) | 31 (2.59) |  |
| Weekly | 849 (7.66) | 41 (3.42) |  |
| Daily | 905 (8.17) | 40 (3.34) |  |
| **Watching TV and/or listening to the radio** |  |  | <0.001 |
| Rarely or never | 2,041 (18.42) | 494 (41.20) |  |
| Sometimes | 552 (4.98) | 69 (5.75) |  |
| Monthly | 466 (4.21) | 71 (5.92) |  |
| Weekly | 1,426 (12.87) | 142 (11.84) |  |
| Daily | 6,596 (59.53) | 423 (35.28) |  |
| **Social activities (organized)** |  |  | <0.001 |
| Rarely or never | 9,005 (81.27) | 1,083 (90.33) |  |
| Sometimes | 873 (7.88) | 49 (4.09) |  |
| Monthly | 466 (4.21) | 26 (2.17) |  |
| Weekly | 362 (3.27) | 23 (1.92) |  |
| Daily | 375 (3.38) | 18 (1.50) |  |
| **Hypertension** |  |  | 0.002 |
| No | 8,969 (80.94) | 1,016 (84.74) |  |
| Yes | 2,112 (19.06) | 183 (15.26) |  |
| **Diabetes** |  |  | 0.002 |
| No | 10,802 (97.48) | 1,187 (99.00) |  |
| Yes | 279 (2.52) | 12 (1.00) |  |
| **Stroke, cerebrovascular disease** |  |  | 0.339 |
| No | 10,555 (95.25) | 1,150 (95.91) |  |
| Yes | 526 (4.75) | 49 (4.09) |  |
| **Parkinson’s disease** |  |  | 0.146 |
| No | 11,049 (99.71) | 1,192 (99.42) |  |
| Yes | 32 (0.29) | 7 (0.58) |  |

| Supplemental Table 2. Best parameters for machine learning models | |
| --- | --- |
| **Parameters** | **Value** |
| **Logistic Regression** | |
| C | 19306.977 |
| penalty | L2 |
| solver | liblinear |
| **SVM** | |
| C | 100 |
| gamma | 0.0001 |
| Kernel | RBF |
| **Random Forest** | |
| criterion | Gini index |
| max_depth | 4 |
| max_features | 5 |
| min_impurity_decrease | 0 |
| min_samples_leaf | 37 |
| min_samples_split | 2 |
| min_weight_fraction_leaf | 0 |
| n_estimators | 100 |
| bootstrap | True |
| **LightGBM** | |
| boosting_type | GBDT |
| colsample_bytree | 1.0 |
| learning_rate | 0.1 |
| max_depth | 4 |
| min_child_samples | 20 |
| min_child_weight | 0.001 |
| min_split_gain | 0 |
| n_estimators | 100 |
| num_leaves | 5 |
| reg_alpha | 1e-05 |
| reg_lambda | 0.5 |
| subsample | 1.0 |
| min_data_in_leaf | 490 |
| bagging_fraction | 0.9 |
| bagging_freq | 3 |
| feature_fraction | 0.7 |
| **XGBoost** | |
| colsample_bytree | 0.6 |
| gamma | 0.1 |
| learning_rate | 0.05 |
| max_depth | 3 |
| min_child_weight | 6 |
| n_estimators | 100 |
| reg_alpha | 3 |
| reg_lambda | 2 |
| subsample | 0.8 |
| scale_pos_weight | 10.558 |
| **MLP** | |
| hidden layer size | 16 |
| loss | BCE Loss |
| optimizer | Adam |
| learning rate | 0.01 |
| momentum | 0 |
| activation function | ReLU |
| output function | Sigmoid |
| training epoch | 300 |
| **Stacking** | |
| meta_classifier | Logistic Regression |
| LR_C | 0.001389 |
| LR_penalty | L2 |
| LR_solver | liblinear |
| use_probas | True |

| Supplemental Table 3. Confusion matrix for each model. | | |
| --- | --- | --- |
| **LR** | Predicted negative | Predicted positive |
| True negative | 5,117 | 1,782 |
| True positive | 163 | 502 |
| **SVM** | Predicted negative | Predicted positive |
| True negative | 5,012 | 1,887 |
| True positive | 153 | 512 |
| **RF** | Predicted negative | Predicted positive |
| True negative | 4,435 | 1,887 |
| True positive | 116 | 549 |
| **LGB** | Predicted negative | Predicted positive |
| True negative | 4,810 | 2,089 |
| True positive | 133 | 532 |
| **Xgboost** | Predicted negative | Predicted positive |
| True negative | 4,999 | 1,900 |
| True positive | 155 | 510 |
| **MLP** | Predicted negative | Predicted positive |
| True negative | 5,245 | 1,654 |
| True positive | 181 | 484 |
| **Fusion** | Predicted negative | Predicted positive |
| True negative | 4,970 | 1,929 |
| True positive | 151 | 514 |

| Supplemental Table 4. The association of selected factors with cognitive impairment. | | | |
| --- | --- | --- | --- |
| **Variable** | **N (%)** | **OR (95% CI)** | ***P* value** |
| **Gender** |  |  |  |
| Female | 11,749 (51.26) | REF |  |
| Male | 11,171 (48.74) | 0.73 (0.64, 0.82) | <0.001 |
| **Age group, year** |  |  |  |
| ~79 | 9,109 (39.74) | REF |  |
| 80~89 | 7,105 (31.00) | 2.06 (1.78, 2.38) | <0.001 |
| 90~99 | 5,019 (21.90) | 4.27 (3.73, 4.87) | <0.001 |
| 100~ | 1,687 (7.36) | 6.73 (5.76, 7.86) | <0.001 |
| **Education, year** |  |  |  |
| Illiterate | 11,867 (51.78) | REF |  |
| 1-6 | 7,938 (34.63) | 0.66 (0.58, 0.77) | <0.001 |
| ≥ 7 | 3,115 (13.59) | 0.60 (0.47, 0.77) | <0.001 |
| **ADL** |  |  |  |
| No ADL | 20,910 (91.23) | REF |  |
| ADL | 2,010 (8.77) | 1.25 (1.10, 1.44) | 0.001 |
| **Garden works** |  |  |  |
| Rarely or never | 18,594 (81.13) | REF |  |
| Yes | 4,326 (18.87) | 0.75 (0.63, 0.89) | 0.001 |
| **Reading newspapers or books** |  |  |  |
| Rarely or never | 16,826 (73.41) | REF |  |
| Yes | 6,094 (26.59) | 0.80 (0.67, 0.97) | 0.023 |
| **Playing Majong or cards** |  |  |  |
| Rarely or never | 21,047 (91.83) | REF |  |
| Yes | 1,873 (8.17) | 0.69 (0.53, 0.90) | 0.006 |
| **Watching TV or listening to the radio** |  |  |  |
| Rarely or never | 18,579 (81.06) | REF |  |
| Yes | 4,341 (18.94) | 0.72 (0.64, 0.80) | <0.001 |
| **Baseline MMSE** |  | 0.90 (0.89, 0.90) | <0.001 |
